# Bilateral Deep Brain Stimulation of the Subthalamic Nucleus under Sedation with Propofol and Fentanyl

**DOI:** 10.1371/journal.pone.0152619

**Published:** 2016-03-28

**Authors:** Woong-Woo Lee, Gwanhee Ehm, Hui-Jun Yang, In Ho Song, Yong Hoon Lim, Mi-Ryoung Kim, Young Eun Kim, Jae Ha Hwang, Hye Ran Park, Jae Min Lee, Jin Wook Kim, Han-Joon Kim, Cheolyoung Kim, Hee Chan Kim, Eunkyoung Park, In Young Kim, Dong Gyu Kim, Beomseok Jeon, Sun Ha Paek

**Affiliations:** 1 Department of Neurology, Eulji General Hospital, Seoul, Republic of Korea; 2 Department of Neurology, Myongji Hospital, Gyeonggi, Republic of Korea; 3 Department of Neurology, Ulsan University Hospital, Ulsan, Republic of Korea; 4 Medical Device Development Center, Osong Medical Innovation Foundation, Chungcheong, Republic of Korea; 5 Department of Neurosurgery, Seoul National University Hospital, Seoul, Republic of Korea; 6 Department of Neurology, Hallym University Sacred Heart Hospital, Gyeonggi, Republic of Korea; 7 Department of Neurosurgery, Daejeon Woori Hospital, Gyeonggi, Republic of Korea; 8 Department of Neurology, Seoul National University Hospital, Seoul, Republic of Korea; 9 Medical Imaging Laboratory, CyberMed Inc., Seoul, Republic of Korea; 10 Department of Medical Engineering, Seoul National University College of Medicine, Seoul, Republic of Korea; 11 Department of Biomedical Engineering, Han Yang University, Seoul, Republic of Korea; 12 Department of Neurosurgery, Seoul National University College of Medicine, Seoul, Republic of Korea; 13 Department of Neurology, Seoul National University College of Medicine, Seoul, Republic of Korea; UCLA, UNITED STATES

## Abstract

Awakening during deep brain stimulation (DBS) surgery may be stressful to patients. The aim of the current study was to evaluate the effect on MER signals and their applicability to subthalmic nucleus (STN) DBS surgery for patients with Parkinson’s disease (PD) under sedation with propofol and fentanyl. Sixteen consecutive patients with PD underwent STN-DBS surgery with propofol and fentanyl. Their MER signals were achieved during the surgery. To identify the microelectrodes positions, the preoperative MRI and postoperative CT were used. Clinical profiles were also collected at the baseline and at 6 months after surgery. All the signals were slightly attenuated and contained only bursting patterns, compared with our previous report. All electrodes were mostly located in the middle one third part of the STN on both sides of the brain in the fused images. Six months later, the patients were improved significantly in the medication-off state and they met with less dyskinesia and less off-duration. Our study revealed that the sedation with propofol and fentanyl was applicable to STN-DBS surgery. There were no significant problems in precise positioning of bilateral electrodes. The surgery also improved significantly clinical outcomes in 6-month follow-up.

## Introduction

Deep brain stimulation (DBS) of the subthalamic nucleus (STN) is helpful in advanced Parkinson’s disease (PD) patients with motor fluctuation and levodopa-induced dyskinesia.[1] Precise localization of the electrode contacts on the STN is very important to achieve the best clinical outcome and to avoid various complications from the stimulation of surrounding structures.[2]

There are several methods to achieve exact localization of the STN. Stereotactic surgery with visualized brain imaging is a representative way to localize the target. However, intraoperative brain shift by CSF leakage and position change could cause some trouble.[3] To solve this problem, conscious patients are evaluated during the operation.[4] This could be a good method in terms of early detection of effects and complications from DBS. Microelectrode recording (MER) is another method to reach the target using specific signals.[5] At first, it was used as a supportive procedure. Most surgeons simulate targeting with brain MRI, approach the STN with MER, and then check for improvements and complications with intraoperative macrostimulation.[6, 7]

However, intraoperative awakening may be stressful in patients. Thus, there have been several attempts to investigate whether proper positioning of the electrodes could be verified under sedation or general anesthesia. Previously, we also investigated the possibility of DBS surgery without intraoperative awakening.[8] An approach with MER and macrostimulation was applied to the left side of the brain, and a method using MER while the patient was under sedation was performed on the right side of the brain. Even though a change in MER signals was unavoidable like in previous studies,[9–13] there were no problems locating the STN and no adverse effects with similar clinical outcomes for both sides of the brain.

The aim of the current study was to assess the effect on MER signals and their applicability to STN DBS surgery when both sides of the brain are under sedation with propofol and fentanyl.

## Methods

The study protocol was approved by the Institutional Review Board of Seoul National University Hospital (IRB No. 1503-124-658) which takes charge of the ethical review. This study was conducted adhering to the declaration of Helsinki. The written informed consents for clinical records were not achieved by the subjects. Because the study was performed retrospectively and the records of patients were anonymized and de-identified prior to analysis. All the clinical evaluations which were described in the methods have been conducted routinely for all the PD patients with DBS surgery.

### Subjects

Seventeen advanced PD patients under sedation with propofol and fentanyl underwent bilateral STN DBS surgery between October 2011 and Aug 2012. All of them were diagnosed with the UK PD Society Brain Bank criteria.[14] One patient was excluded from the analysis because of a previous history of pallidotomy. Finally, 16 patients were enrolled in the current study.

We reviewed the medical records of all cases retrospectively. Intraoperative MER data were analyzed for both sides of the brain for all 16 patients. The patients were examined with the Unified Parkinson Disease Rating Scale (UPDRS), Hoehn and Yahr (H&Y) Staging, Schwab and England Activities of Daily Living (SEADL), the Short Form-36 Health Survey (SF-36), and neuropsychological tests. Evaluations were performed before surgery and 6 months after surgery. The neurological examinations were done by experienced neurologists. Patients were assessed under two conditions (off medication when the patients had taken no medication for 8 to 12 hours and on medication when the patients had experienced maximal clinical benefit 1 to 3 hours after the usual morning dose of dopaminergic treatment) before and after surgery. The levodopa equivalent daily dose (LEDD) was computed as previously described.[15]

### Perioperative procedure

Anti-Parkinsonian medications were not stopped preoperatively because we did not awaken the patient during the operation to assess the response to macrostimulation. Other surgical procedure was similar to our previous study.[8] A stereotactic Leksell^®^-G frame (Elekta Instruments AB, Stockholm, Sweden) was mounted on the head under local anesthesia. A brain MRI with 1.5T system was taken. (General Electric Medical System, Milwaukee, WI, U.S.A.) The FSPGR 3-D sequence was used for anterior commissure (AC)–posterior commissure (PC) calculations. T2 spin-echo images were obtained to define the boundaries of the STN. SurgiPlan^®^ (Eleckta, Stockholm, Sweden) conducted the simulations for targeting the sensorimotor region of the STN and selecting the trajectories. The operations were performed under monitored anesthesia care (MAC) with continuous infusion of propofol (25 ug/kg/min) and fentanyl (25 ng/kg/min). The depth of sedation was monitored by the bispectral index and also estimated by the degree of the patients’ awakening response to a loud sound and shaking as well as the response to pinching. The characteristic discharges of the bilateral STN were identified using MER by LeadPoint (Medtronic, Minneapolis, MN). The permanent quadripolar electrodes were implanted along the proper trajectory to stimulate more sensorimotor region of the STN. The STNs were localized by a combination of brain MRI and intraoperative MER. We did not use an intraoperative macrostimulation technique.[15] The stereotactic frame was removed and the implantable pulse generators (IPG) (Medtronic, Minneapolis, MN) were implanted in a subcutaneous pocket below both clavicles under general anesthesia in a single session.

Electrical stimulation was started one day after surgery. The patients also took medications but at a reduced dose compared to their previous dose. The medications and stimulation parameters were progressively adjusted using an N’vision® programmer (Medtronic, Minneapolis, MN) according to their clinical status.[15]

3-D spiral stereotactic CT scans (64-channel Brilliance CT, Philips, Eindhoven, Netherlands) with a 1- mm slice thickness were immediately taken postoperatively and one month after bilateral STN stimulation to localize the electrodes by image fusion with the preoperative MRI using mutual information techniques as previously described.[15] With CT-MRI image fusion, the electrodes positions were plotted on the human brain atlas of Schaltenbrand and Wahren.[16]

### Microelectrode Recordings

We used the same protocol as previously.[8] The MER signals along the selected trajectories were collected for analysis. Each MER signal was band-pass filtered at 500–5,000 Hz with a gain 10,000. The sampling frequency was 24 kHz. A threshold was applied at 3 SD over the background noise in the intraoperatively recorded spontaneous neuronal activity and this threshold was applied for spike sorting. Spike sorting for a single unit was performed using the Offline Sorter software (Offline Sorter, Plexon, TX, USA). Principal components were calculated for unsorted waveforms, and the waveforms were assigned to clusters using the expectation-maximization algorithm based on the T-distribution method. Two statistical parameters, the J3 statistic and the Davies-Bouldin (DB) validity, were used to examine the sorting quality statistics between classified clusters. A high value for the J3 statistic and a low value for the DB validity indicates that the clusters are compact and well-separated. Single unit activity was classified as non-burst (tonic, irregular), or as a burst discharge pattern using the method of Kaneoke and Vitek.[17] The mean inter-spike interval was used for making spike train, and the distribution of discharge densities was assessed whether it follows a Poisson distribution. Burst pattern means that the distribution of discharge densities on the spike train meets neither a normal nor a Poisson distribution and is positively skewed. Non-burst pattern is the collective name of regular pattern and random pattern. The discharge density distribution of regular pattern follows normal distribution and it of random pattern meets a Poisson distribution.

### Statistical analysis

The data for the aforementioned variables were presented as the mean ± standard deviation. To examine the effect of anesthesia and the pattern on the mean firing rate and the effect of different combinations of anesthesia and the pattern on the mean firing rate, analysis of variance (ANOVA) was done.

Paired/unpaired t-tests and Fisher’s exact test were used as appropriate for the comparison between the baseline before surgery and 6 months after surgery. P-values of 0.05 were considered to indicate statistical significance. All statistical analyses were done with the SAS statistical software (Version 9.0).

## Results

All 16 subjects (12 women, and 4 men) were included in the analysis. The mean age at DBS surgery was 57.7 ± 6.2 years, and the mean duration from onset to surgery was 12.6 ± 5.6 years. Baseline UPDRS, HY, ADL, and LEDD are presented in Table 1. These data were not different from those in our previous studies.[8, 15]

**Table 1:** Clinical Outcome of bilateral subthalamic nucleus deep brain stimulation under sedation.

	Medication	Baseline	6 months after surgery	*p* value
**UPDRS part I**	On	2.2 ± 1.8	1.8 ± 1.9	0.473
	Off	5.0 ± 3.6	3.9 ± 2.7	0.270
**UPDRS part II**	On	10.1 ± 8.0	9.2 ± 7.7	0.329
	Off	29.2 ± 9.0	18.8 ± 9.3	0.002**
**UPDRS part III**	On	22.6 ± 8.5	17.5 ± 10.4	0.092
	Off	50.0 ± 12.1	23.3 ± 8.2	< 0.001***
**Total UPDRS**	On	34.9 ± 15.4	28.5 ± 17.5	0.215
	Off	84.2 ± 18.3	36.4 ± 13.7	< 0.001***
**Proportion of dyskinesia period ^a^ (%)**		52.0 ± 28.9	14.7 ± 28.2	0.021*
**Proportion of off period ^a^ (%)**		51.6 ± 20.9	31.3 ± 18.5	0.020*
**H&Y**	On	2.4 ± 0.4	2.5 ± 0.7	0.617
	Off	3.4 ± 0.6	2.6 ± 0.5	< 0.001***
**ADL**	On	74.4 ± 24.5	83.1 ± 16.2	0.273
	Off	31.3 ± 24.5	63.8 ± 23.1	< 0.001***
**LEDD (mg/d)**		1593.7 ± 443.3	662.1 ± 297.1	< 0.001***
**MMSE**		27.1 ± 2.5	25.3 ± 3.9	0.038*
**BDI**		23.7 ± 7.5	20.2 ± 11.5	0.219
**SF36-Physical Health**		122.6 ± 62.0	183.9 ± 72.8	0.007**
**SF36-Mental Health**		131.4 ± 87.2	192.3 ± 83.3	0.004**

UPDRS, Unified Parkinson’s Disease Rating Scale; H&Y, Hoehn and Yahr scale; ADL, Activities of Daily Life; LEDD, Levodopa equivalent daily dose; MMSE, Mini-mental state examination; BDI, Beck Depression Inventory; SF 36, Short Form-36

^a^ Hours of dyskinesia divided by total waking hours per day

^b^ Hours of off symptom divided by total waking hours per day

* p<0.05

** p<0.01

*** p<0.001

### MER Analysis

**Fig 1:**
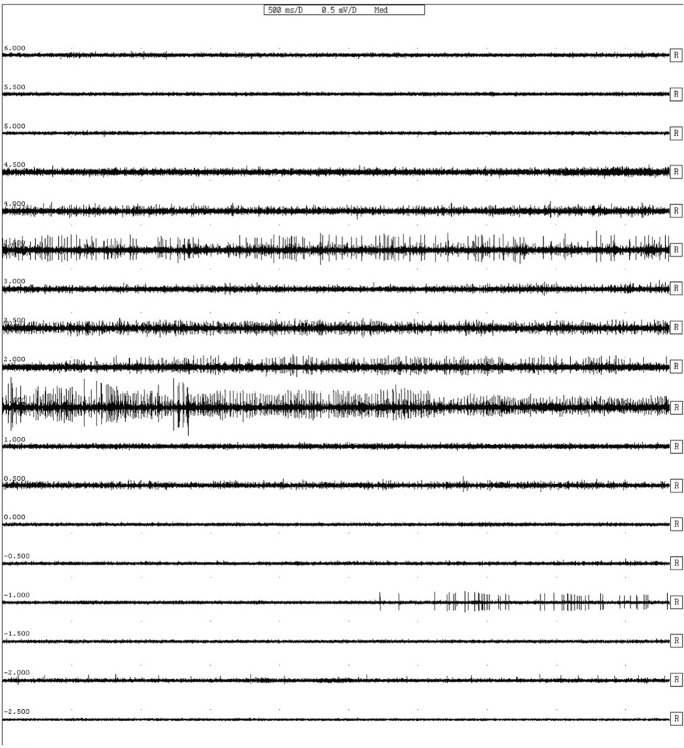
Typical bursting patterns from microelectrode recording.

**Fig 2:**
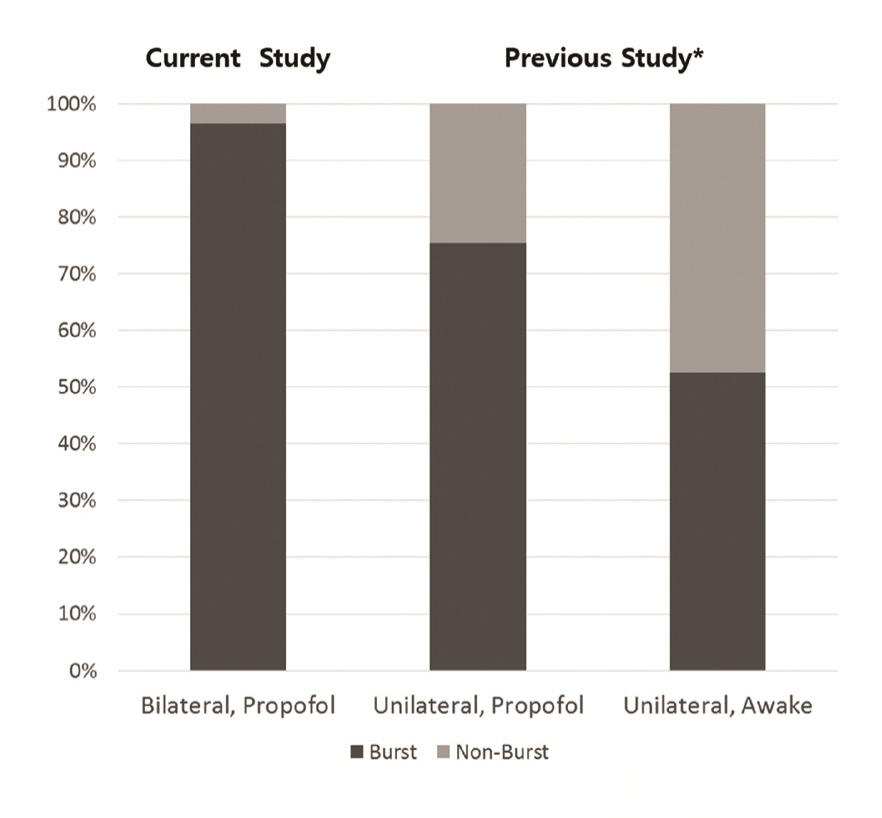
The proportion of burst and non-burst discharges. *Kim et al., 2014^8^.

Typical STN bursting patterns appeared on both sides of the brain,[5] although the background signal noise was decreased, compared to our previous report.[8] (Fig 1) Six snoring events (2 events for right side and 4 events for left side) occurred among 32 MER procedures for all patients. Whereas there were no remarkable problems in visual assessment of STN signals with snoring, (S1 Fig) they could not be included in the signal analysis. The total number of sampled neurons was 57, the firing rate was 19.6 ± 13.5 spikes/sec, and the inter-spike interval of burst period was 12.3 ± 5.7 ms. The proportion of bursting pattern was 96.5% which was higher in the current study than in the previous report.[8] (Fig 2) The MER profiles of each side were described in Table 2.

**Table 2:** Characteristics of signals on microelectrode recording.

	The total number of single unit activities	The proportion of bursts (%)	The firing rate (spikes/sec)	The inter-spike interval of burst period (ms)
**Right STN ^a^**	24	100	17.4 ± 12.1	13.6 ± 4.8
**Left STN ^a^**	33	93.9	21.3 ± 14.2	11.3 ± 6.0

STN, subthalamic nucleus

^a^ The MER data on snoring were excluded in the signal analysis: 2 events for right side and 4 events for left side.

### Electrode Position after bilateral STN-DBS

**Fig 3:**
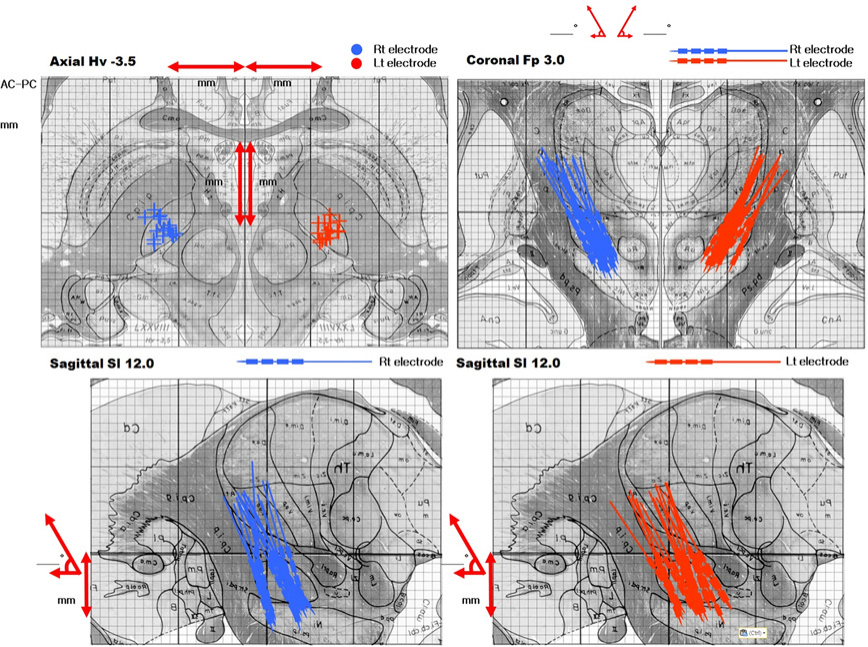
The location of electrodes on brain atlas. Based on the CT-MRI fusion images of the preoperative brain MRI and postoperative brain CT scan taken one month after surgery. The figure shows location of the electrodes plotted onto the human brain atlas of Schaltenbrand and Wahren.

The bilateral electrodes were inserted into the STNs depending on the bursting signals from MER. To identify whether each inserted electrodes was in its targeted structure, the microelectrodes positions were plotted in the sagittal and coronal planes by the AC-PC line according to the CT findings 1 month after the operation. All the electrode positions were mostly located in the middle one third part of the STN on both sides of the brain in the fused images, which was confirmed from the reformatted images of the fused images between the preoperative MRI and postoperative CT. (Fig 3)

### Clinical Outcome 6 months after bilateral STN-DBS

The clinical changes after STN-DBS are summarized in Table 1. Above all, there were significant improvements in clinical scales when the patients had not taken any medications. The change in score for the UPDRS part I in the medication-off state did not reach a significant level. Total UPDRS, UPDRS part I/II/III, H&Y, and ADL at the on-time also did not show any significant changes, even though they showed trends for improvement. The relative daily time of dyskinesia or off symptoms was reduced significantly. (52.0 ± 28.9 to 14.7 ± 28.2%, for dyskinesia; 51.6 ± 20.9 to 31.3 ± 18.5%, for off-time) The MMSE score was slightly aggravated, but significant improvements in the SF-36 scores was observed 6 months later. There were 2 cases of transient apathy postoperatively and 2 cases of wound infection which were improved by antibiotics and wound revision.

## Discussion

The clinical outcome of STN-DBS depends on various factors.[5] Among them, optimal electrode positioning is a very important factor. It can lead to good results or irreversible complications. Thus, most neurosurgeons have tried to use all means available to identify the STN. Intraoperative macrostimulation is one of the very reliable methods available to an operator.[5] However, it could be also a stressful time for patients because patients have to stop taking anti-parkinsonian drugs before surgery and endure awakening during the surgery. If STN-DBS can be performed precisely without intraoperative macrostimulation, it will be better all-around for the patient and the operator.

To approach the STN only with the preoperative stereotactic method is nearly impossible without intraoperative macrostimulation and MER because there are intraoperatively uncorrectable issues such as brain shift due to CSF leakage and errors in the manipulation of MRIs.[3, 18–20] Although intraoperative accuracy evaluations using MRI or CT have recently been developed as an alternative, there are some unsolved problems.[21–24] Intraoperative imaging tools are not promptly interactive, and most hospitals are not equipped with them. Above all, more evidence is needed. In contrast, MER can provide prompt feedback to the operator. The preparation of MER is not difficult as that of intraoperative imaging tools.

Propofol is a sedative that can be used to alleviate anxiety and unpleasant memories. However, the MER signals of the STN are inevitably affected by sedatives including propofol.[25] To ascertain whether the changes in MER signals are clinically critical, we performed this study in stages: 1) Was the MER signal changed by propofol; 2) Did the change in MER signal affect precise targeting of the STN, and 3) Was the clinical outcome good without intraoperative macrostimulation?

The proportion of non-bursting discharge and the firing rate were also more decreased than those when the procedures under propofol and fentanyl had been done for the unilateral side in our previous report.[8] The single unit STN neurons in the present study showed a bursting pattern in 96.5%, whereas in the previous study, 75.4% (under sedation with propofol and fentanyl) and 52.6% (under local anesthesia) exhibited a bursting discharge. The mean firing rates also had a similar pattern to the proportions of the bursting discharge (19.6 spikes/sec, with sedation in the current study; 35.5 spikes/sec, with sedation in the previous study; 38.7 spikes/sec, with local anesthesia in the previous study). The differences in the firing rate and the bursting proportion between 2 studies may be caused by whether the patients took anti-parkinsonian medications or not. In the current study, anti-parkinsonian medications were not discontinued. It probably contributed to the decrease of firing rate and the dominance of bursting pattern because STN lies on indirect pathway of cortico-basal ganglia-thalamo-cortical circuit. In addition, the total number of sampled neurons was small. It means that the neuronal activity of STN may be underestimated and cell selection bias may affect the results. Another possible explanation is that the longer duration of sedation might influence the attenuation of MER signals. In the previous study, we applied first local anesthesia for the left side procedure and then sedated with propofol and fentanyl for the right side procedure. This graduated trend would be determined by the depth of sedation.

For the features of the MER signal, these differences could be relatively big compared with previous reports.[7, 8, 26–31] However, the final purpose of MER is not just to achieve a signal pattern but to reach a precise target. If the changed signal does not affect the localization of the STN, it is not a problem clinically. Therefore, we postoperatively checked the locations of the electrodes. Actually, all the electrodes were well-located within the STN as in our previous report[8] even though the MER signals were slightly attenuated.

Besides, the STN-DBS operations were successful in terms of clinical outcomes. The UPDRS II/III, H&Y, and ADL all showed meaningful changes when the patients were in the off state. The decreases in UPDRS II and UPDRS III scores between baseline and 6 months were 35.6% (29.2 → 18.8) and 53.4% (50.0 → 23.3) respectively. We already reported 41.9% [32] and 55.7% [15] improvements of UPDRS III scores in our previous studies with macrostimulation. One comprehensive review[5] also showed comparable results (27% to 72.6% for UPDRS II, 28% to 71% for UPDRS III) with the current study. The remarkable decrease in LEDD was also similar to previous studies.[5, 15, 32] In addition, there were trends showing that all these scales were improved even for the medication-on state. The patients could have a longer on-time with less dyskinesia and a better quality of life in terms of physical and mental health.

There was a mild decline in cognitive function. The reason of cognitive impairment is not clear. Postoperative cognitive dysfunction due to anesthesia could be a possible cause,[33] but our previous study which underwent awake surgery showed cognitive decline postoperatively.[15] The withdrawal of medications, the lesion effects, and the stimulation of limbic or associative regions of the STN also should be considered. A few postoperative complications became improved during the follow-up.

There already have been several studies on the usefulness of propofol in STN-DBS.[9, 26, 34–40] The majority of them have reported on the clinical effectiveness of STN-DBS even under sedation with propofol. Our study has also revealed similar clinical results to that of previous studies.[8] The big difference between our and other studies is that we confirmed the location of the electrodes with a fusion image as well as the patient’s clinical status. Our results provide concrete evidence that MER is helpful in providing exact targeting. This study has several limitations. First, the scale of this study is not large, and the analysis was performed retrospectively. Second, the data covered only a 6 month follow-up period. A further prospective and well-controlled study can improve the significance of the evidence by including a larger population and long-term follow-up.

## Conclusions

The MER signals of bilateral STNs under propofol were slightly attenuated. However, the changes in the MER signals did not interfere with targeting good locations in the postoperative fusion images. Additionally, all the included patients improved clinically without any remarkable complications, and the clinical outcomes were improved without stressing the patients. In conclusion, propofol and fentanyl can be used safely for bilateral STN-DBS surgery in advanced PD patients.

## Supporting Information

S1 Fig Typical bursting patterns under snoring. (TIF)

S1 File Clinical and MER data of the present study. (XLSX)
