# Supplementary figures and images for: Bilateral Deep Brain Stimulation of the Subthalamic Nucleus under Sedation with Propofol and Fentanyl
Source: PLoS One. 2016 Mar 28;11(3):e0152619. doi: 10.1371/journal.pone.0152619 (PMC4809591; doi:10.1371/journal.pone.0152619)

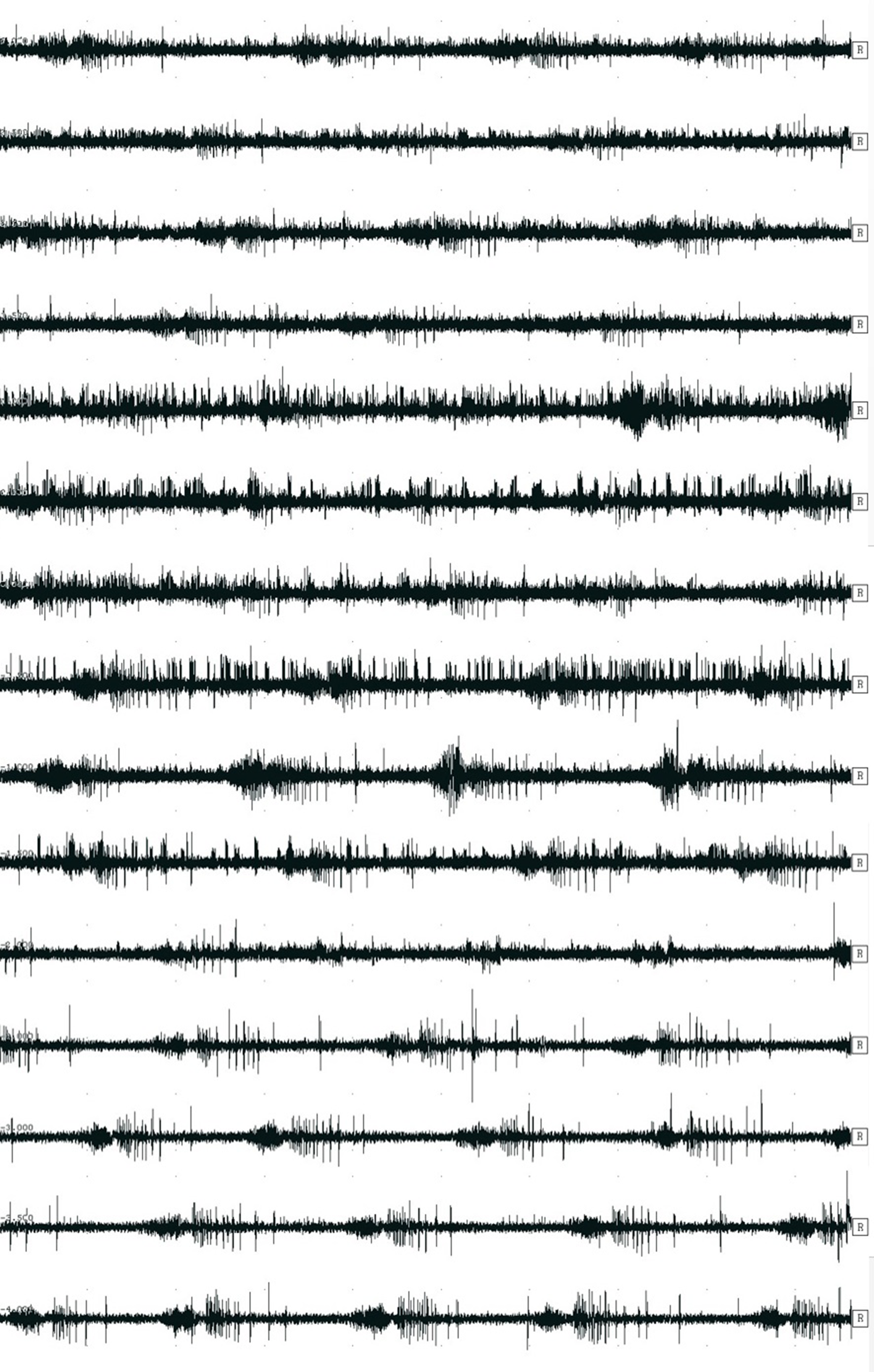

Supplement: S1 Fig — (TIF) [file pone.0152619.s001.tif]
